# Immune imbalance markers: key factors in early recognition of multidrug-resistant bacterial infections in non-immunocompromised VAP patients

**DOI:** 10.3389/fimmu.2026.1799042

**Published:** 2026-06-04

**Authors:** Mingying Tang, Qiyong Meng, Zhimin Huang, Yongjun Qing, Weijian Lei

**Affiliations:** 1 Department of Intensive Care Unit, The Affiliated Qingyuan Hospital (Qingyuan People’s Hospital), Guangzhou Medical University, Qingyuan, Guangdong, China; 2 Guangzhou Medical University, Guangzhou, China

**Keywords:** biomarker, cytokine, immune imbalance, multidrug-resistant organism, severe neurological disorders, ventilator-associated pneumonia, interleukin-6, interleukin-10

## Abstract

**Background:**

Research on the role of immune dysregulation in multidrug-resistant organism (MDRO) infections among patients with ventilator-associated pneumonia (VAP) remains limited, and there is a lack of reliable immunological indicators for early identification.

**Methods:**

We conducted a retrospective analysis of 247 non-immunocompromised patients who developed VAP following mechanical ventilation in the Neurointensive Care Unit (NICU). Patients were categorized into MDRO and non-MDRO groups based on the presence or absence of MDRO infection. Binary logistic regression with sequential adjustment (three models), subgroup analyses, and receiver operating characteristic (ROC) curve analysis were used to evaluate associations between MDRO infection and early-stage (within 72 hours) immunological indicators, including IL-10, IL-17A, IL-6, and T-cell parameters (CD4^+^, CD8^+^, CD3^+^ counts, and CD4/CD8 ratio). Additionally, six pathogen-specific subgroups were analyzed to assess differences in immune factor levels across pathogen types.

**Result:**

In univariate analysis, T-cell parameters showed no statistical significance, but IL-10 and IL-6 levels were significantly elevated in the MDRO group. In the fully adjusted logistic regression model, IL-10 and IL-6 were identified as independent risk factors. Higher IL-10 tertiles showed a dose-response relationship with MDRO risk. Subgroup analysis indicated that elevated IL-10 and IL-6 increased MDRO risk in patients with albumin ≥30 g/L. ROC analysis demonstrated good diagnostic performance for IL-10, IL-6, and their combined model (IL-10+IL-6). Serum IL-6 levels were significantly higher in patients with extended-spectrum beta-lactamase-producing Klebsiella pneumoniae (ESBL-KP) infection than in those with carbapenem-resistant Acinetobacter baumannii (CRAB) infection, indicating pathogen specificity. In contrast, IL-10 levels did not differ significantly across MDRO subgroups (CRAB, ESBL-KP, CRPA, and mixed infection), suggesting a universal host immune response to MDRO infection.

**Conclusions:**

Within 72 hours after VAP onset, elevated serum IL-10 and IL-6 levels are an immune dysregulation state resulting from multiple immune factor imbalance mechanisms, which may facilitate early identification of patients at risk for MDRO infection, with serum IL-10 levels showing potential value as an independent biomarker. Serum IL-10 levels may sensitively reflect the host’s immune dysregulation status, whereas serum IL-6 levels may indicate a specific immune response to a certain MDRO strain.

## 1 Introduction

Ventilator-associated pneumonia (VAP) is a particularly common hospital-acquired infection (HAI) in neurointensive care units (NICUs). According to a global report from the International Nosocomial Infection Control Consortium (INICC) spanning 123 cities, the incidence of VAP is highest in trauma and neurologic intensive care units, at 11.96 episodes per 1000 ventilator-days, with a mortality rate reaching 36.89% (1). Moreover, patients with VAP in the NICU have a higher probability of multi-drug resistant organism (MDRO) infection compared to those in other ICUs or general wards (2). This leads to adverse clinical outcomes, including the frequent use of broad-spectrum antibiotics, difficulty in weaning from mechanical ventilation, and impaired neurological recovery (3, 4).

Patients in the NICU are at high risk for MDRO infection in VAP (5–7). This susceptibility stems from frequent immune dysfunction and impaired respiratory defense mechanisms, which are associated with primary neurological conditions (e.g., traumatic brain injury, stroke) (8, 9), surgical interventions, nutritional and metabolic disturbances, use of broad-spectrum antibiotics and corticosteroids, prolonged bed rest, and immobilization. Traditional serum inflammatory biomarkers offer limited utility in current clinical practice due to their low sensitivity and specificity (10), highlighting the need to explore novel indicators of immune dysregulation. Immune imbalance is an early manifestation and central mechanism of immune dysfunction during bacterial infection (11, 12). It involves a disruption of immune homeostasis (13), driven by factors such as an imbalance between pro- and anti-inflammatory responses (14, 15), altered ratios of T lymphocytes and their subsets (16, 17), and dysregulated cytokine levels (18–20). These elements, along with dysfunction of innate immune cells, form a complex immunological network. The persistence and accumulation of multiple imbalances can trigger a cytokine storm, leading to systemic immune dysregulation. This state is characterized by immune exhaustion and severely compromised pathogen clearance, contributing to the susceptibility and refractory nature of VAP (21, 22).

Imbalances in T lymphocyte subsets and cytokine levels represent primary manifestations of systemic immune dysregulation. In patients with severe bacterial pneumonia, CD4^+^ T cells, CD8^+^ T cells, and B cells often display an exhausted phenotype, while regulatory T cell (Treg) populations expand. This immune dysregulation suggests an immunosuppressive microenvironment that may impede bacterial clearance (23). Restoring the balance between Th17 and Treg cells helps maintain pulmonary immune homeostasis and protect lung tissue. IL-17A is an early pro-inflammatory cytokine produced by Th17 cells, IL-10 is an anti-inflammatory cytokine characteristic of Treg cells, and IL-6 serves as a major mediator of systemic inflammation. However, individual cytokines provide limited information. Abnormal expression levels of both pro- and anti-inflammatory factors can better reflect the state of immune balance. Studies have suggested that IL-6 and IL-10 can aid in the preliminary differentiation between Gram-negative and Gram-positive bacterial infections (20, 24). Other evidence indicates their association with pneumonia severity and potential utility as indicators of anti-infective treatment efficacy (25, 26). Resistant bacterial strains are more likely to emerge, accumulate, and persist in immunosuppressed hosts. For instance, immunosuppression, such as neutropenia, may promote the development of resistant variants in murine pneumonia models, although outcomes following pathogen exposure in the community remain uncertain (27). It is worth noting that the host immune response against specific pathogens has shown potential value in diagnosing VAP infection (28), identifying different stages of infection (29, 30), and even discriminating specific pathogenic strains (31). Building on this evidence, the present study aims to explore indicators of immune dysregulation, specifically the changes in immunological profiles associated with MDRO development in VAP. This study aims to enhance comprehension of immune mechanisms related to infection, identify potential new immune targets for therapy, and explore feasible methods based on immune dysregulation-related indicators to facilitate early clinical identification of high-risk populations for MDRO infection.

## 2 Materials and methods

### 2.1 Study design and patient population

**Figure 1:**
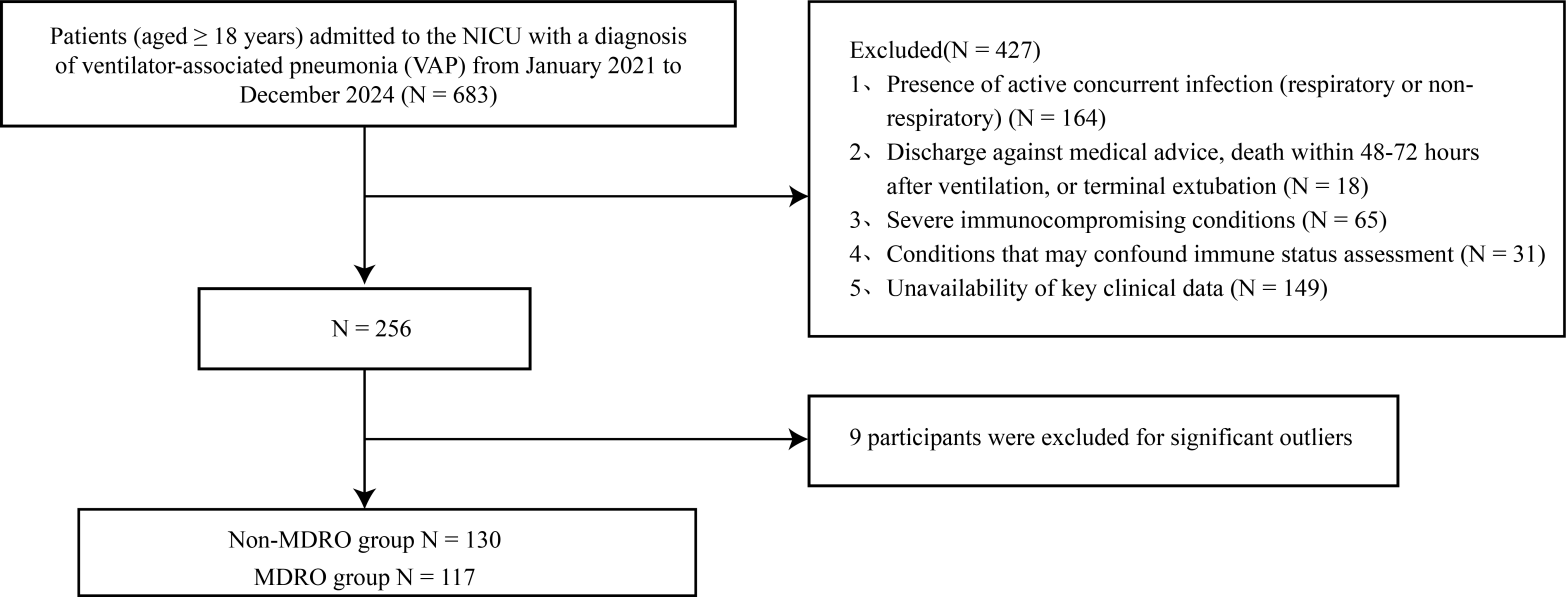
Flowchart depicting the participants’ selection.

Patients were enrolled in this study if they were admitted to the NICU of The Affiliated Qingyuan Hospital (Qingyuan People’s Hospital), Guangzhou Medical University between January 2021 and December 2024, diagnosed with VAP, aged 18 years or older, and had complete clinical data. Patients were excluded under the following conditions: 1) presence of active infection in the respiratory or other systems prior to mechanical ventilation; 2) patients who were discharged against medical advice or died within 48–72 hours after mechanical ventilation, or those who underwent terminal extubation and withdrew from active treatment; 3) comorbid conditions that severely impair immune function, such as autoimmune diseases, HIV infection, tuberculosis, malignancies, or severe hematological disorders; 4) diseases that may complicate the immune status, including cholelithiasis, choledocholithiasis, or severe renal dysfunction requiring hemodialysis; 5) Use of immunosuppressants or corticosteroids within one week before mechanical ventilation. A total of 247 patients were ultimately included in the final analysis (**Figure 1**).

### 2.2 Data Collection and cytokine measurement

A specialized neurointensive care physician retrospectively reviewed the hospital’s electronic medical record system to collect patient data. This included demographic characteristics, medical history, and examination results such as age, sex, diabetes, smoking status, alcohol consumption, surgical status, length of ICU stay, duration of mechanical ventilation, as well as GCS and APACHE II scores from the first day of admission. Prior antibiotic use was defined as any documented prescription for systemic antibacterial agents (ATC code J01) within 90 days before admission. Based on admission records and specialist assessment, the primary neurological disease was defined as the principal neurological disorder leading to the current NICU admission and was coded in accordance with the International Classification of Diseases (ICD–10/11).

Baseline infection and inflammatory burden indicators (complete blood count, albumin, CRP, PCT, and lactate) were collected on day 2 of mechanical ventilation. Blood samples obtained within 72 hours after VAP diagnosis were used to measure IL−6, IL−10, and IL−17A levels (pg/mL) via a human Th1/Th2/Th17 subgroup detection kit (flow cytometry−based immunoassay; Saiji Biotechnology Co., Ltd., Jiangxi, China). The lower detection limits were 2.5 pg/mL for IL−6 and IL−10, and 10 pg/mL for IL−17A. Absolute counts of CD3^+^, CD4^+^, and CD8^+^ T cells (cells/μL) were determined using a BM2000 microscope and an immunocapture assay (CD series cell detection slides; Huizhong Cell Biotechnology Co., Ltd., Shanghai, China), and the CD4/CD8 ratio was calculated. According to the manufacturer’s instructions, the relative deviation of this method compared with flow cytometry results was ≤10%, and the correlation coefficient was ≥0.90. The median time from VAP diagnosis to sample collection for immunological indicators was 2 days (IQR: 1–2 days) in both the MDRO and non−MDRO groups. All tests were performed by the institutional laboratory in strict accordance with standard operating procedures.

### 2.3 Definition of VAP

VAP was clinically diagnosed in patients with >48 hours of mechanical ventilation or within 48 hours of extubation if they presented with new or progressive radiographic infiltrates, consolidation, or ground-glass opacities, plus at least two of the following: (1) fever (>38 °C), (2) purulent airway secretions, (3) WBC count >10 × 10^9^/L or <4 × 10^9^/L.

### 2.4 Definition of MDRO and subgrouping by pathogen type

Following a VAP diagnosis, respiratory specimens, including endotracheal aspirates, bronchoalveolar lavage fluid, or pleural effusion, were collected for microbiological culture. Patients were assigned to the MDRO group if at least one bacterial isolate was cultured and demonstrated concurrent resistance to three or more classes of antimicrobial agents to which it is typically susceptible. Repeated isolation of the same bacterial strain from the same patient was counted as a single case. Samples indicating infection at other sites (e.g., urine, blood, cerebrospinal fluid, wound secretions, or catheter-related samples) were excluded unless the infection was confirmed to have occurred after the VAP diagnosis.

To further analyze the differences in cellular immune factor levels among various drug-resistant bacteria, we excluded pathogen species with small sample sizes (e.g., CRKP, MRSA) and subdivided the MDRO group into four subgroups based on specific pathogens: CRAB (carbapenem-resistant Acinetobacter baumannii), ESBL−KP (extended−spectrum beta−lactamase−producing Klebsiella pneumoniae), CRPA (carbapenem−resistant Pseudomonas aeruginosa), and a mixed MDRO infection group (with two or more MDRO strains detected simultaneously). The non−MDRO group was divided into two subgroups: the non−MDRO culture−positive group (with non−multidrug−resistant bacteria detected) and the non−MDRO culture−negative group (meeting the clinical diagnostic criteria for VAP but with negative pathogen culture).

### 2.5 Statistical analysis

Statistical analyses were performed using SPSS 26.0 and R 4.5.2. Continuous variables were tested for normality (Kolmogorov−Smirnov test, histograms, Q−Q plots). Normally distributed data are presented as mean ± standard deviation and analyzed using independent samples t−test; non−normally distributed data are presented as median (interquartile range) and analyzed using the Mann-Whitney U test. Categorical variables are presented as frequency (percentage) and analyzed using Pearson’s χ² test.

We performed binary logistic regression analysis to evaluate the associations between immunological indicators and MDRO risk. Instead of directly including the variables in the model, stratifying IL-10 and IL-6 by tertiles (T1–T3) allowed us to better demonstrate the dose-response relationship between these immune factors and MDRO infection risk, while avoiding the potential instability that could arise from excessive stratification (e.g., quartiles) and multiple comparisons. We constructed three models for sequential adjustment of confounding factors: Model 1 was unadjusted; Model 2 was adjusted for sex, age, diabetes, smoking status and alcohol consumption; Model 3 was further adjusted for surgical status, primary neurological disease, length of ICU stay, prior antibiotic use, GCS score, APACHE II score, duration of mechanical ventilation, CRP, albumin, PCT and lactate. Compared with building a single model, sequential adjustment allows the dynamic change of effect estimates with sequential control of confounders to be visualized, thereby testing the robustness of the results. Multicollinearity was examined using the variance inflation factor (VIF), with a threshold of VIF < 5. A VIF value of <5 for each variable was considered indicative of no significant multicollinearity. Subgroup analyses stratified by age, albumin and APACHE II scores were performed, including product interaction terms to calculate P for interaction, and forest plots were used to display the effect estimates and their 95% confidence intervals calculated using the profile likelihood method for each subgroup, in order to assess potential effect modification for MDRO infection. ROC analysis was performed to evaluate the predictive performance of immunological indicators for MDRO infection.

Finally, we compared IL-10 and IL-6 levels across six different pathogen subgroups. Because the immune factor data were skewed, overall comparisons among the six groups were performed using the Kruskal-Wallis test. If a statistically significant overall difference was detected, *post-hoc* multiple comparisons among the six groups were subsequently conducted using Dunn’s test. This part represents exploratory analysis. To avoid the overly conservative nature of Bonferroni correction, which could miss genuine biological differences, we chose the Benjamini-Hochberg method to adjust the p-values (false discovery rate, FDR), thereby controlling the FDR in multiple comparisons while preserving adequate statistical power to detect potential intergroup differences.

## 3 Results

### 3.1 Baseline characteristics

Comparison of baseline characteristics revealed no statistically significant differences between the MDRO and non-MDRO groups regarding T-cell parameters (CD4^+^ T cells, CD8^+^ T cells, CD3^+^ T cells, CD4/CD8 ratio), IL-17A levels, age, sex, primary neurological disease, diabetes, or surgical status. However, the MDRO group exhibited more pronounced immune dysregulation and inflammatory response, with significantly elevated levels of IL-10 and IL-6. These patients also had elevated serum lactate [2.00 (1.20–3.20) vs. 1.30 (0.98–3.38) mmol/L, *P* = 0.011], lower serum albumin (30.89 ± 5.80 vs. 32.63 ± 7.44 g/L, *P* = 0.043), higher APACHE II scores (*P* < 0.001), and lower GCS scores (*P* = 0.007), suggesting greater overall severity. Furthermore, the MDRO group had higher smoking rates (*P* = 0.023), more frequent prior antibiotic use (*P* = 0.049), and longer ICU stays and mechanical ventilation durations (*P* < 0.001). Detailed data are shown in **Table 1**.

**Table 1:** Baseline characteristics of patients.

Characteristic	Overall (n=247)	MDRO (n=117)	non-MDRO (n=130)	*p* value
Age, y	60.34 ± 16.46	60.36± 15.88	60.33 ± 17.03	>0.05
Male, n (%)	169(68.4)	83(70.9)	86(66.2)	>0.05
Primary neurological disease				>0.05
Intracerebral hemorrhage, n (%)	107(43.3)	53(45.3)	54(41.5)	
Ischemic stroke, n (%)	43(17.4)	21(17.9)	22(16.9)	
Traumatic brain injury, n (%)	74(30.0)	36(30.8)	38(29.2)	
Other, n (%)	23(9.3)	7(6.0)	16(12.3)	
Diabetes, n (%)	32(13.0)	14(12.0)	18(13.8)	>0.05
Smoking status, n (%)	56(22.7)	34(29.1)	22(16.9)	0.023
Alcohol consumption, n (%)	23(9.3)	14(12.0)	9(6.9)	>0.05
Surgical status, n (%)	153(61.9)	79(67.5)	74(56.9)	>0.05
Prior antibiotic use, n (%)	12(4.9)	9(7.7)	3(2.3)	0.049
Length of ICU stay, Days	13.32 ± 9.85	16.26 ± 10.94	10.67 ± 7.90	<0.001
GCS	6.90 ± 3.53	6.27 ± 3.06	7.46 ± 3.82	0.007
APACHE II score	20.89 ± 6.40	22.66 ± 5.54	19.30 ± 6.71	<0.001
Duration of mechanical ventilation, Days	10.13 ± 7.93	12.76 ± 9.38	7.77 ± 5.38	<0.001
IL-10, pg/ml	4.09(2.30, 6.27)	5.55(3.89, 8.78)	3.11(1.82, 4.42)	<0.001
T1	1.81(1.21, 2.32)	1.92(1.21, 2.45)	1.78(1.26, 2.31)	
T2	4.09(3.54, 4.77)	4.63(3.89, 4.93)	3.87(3.37, 4.37)	
T3	7.96(6.25, 11.25)	8.49(6.53, 11.65)	6.30(5.86, 9.23)	
IL-17A, pg/ml	0.70(0.10, 1.37)	0.78(0.12, 1.72)	0.58(0.08, 1.15)	>0.05
IL-6, pg/ml	37.05(14.79, 94.47)	43.99(15.93, 146.90)	29.39(12.72, 63.57)	0.008
T1	10.00(6.14, 14.79)	10.17(6.68, 14.56)	8.92(5.95, 15.25)	
T2	37.05(26.13, 45.73)	39.19(25.01, 48.16)	34.58(26.93, 45.45)	
T3	150.49(93.51, 340.35)	164.27(101.09, 565.50)	135.09(77.41, 205.03)	
CD4^+^T cells, μL	504.87 ± 292.18	491.69 ± 276.42	516.72 ± 306.26	>0.05
CD8^+^T cells, μL	273.81 ± 167.08	272.28 ± 162.58	275.19 ± 171.65	>0.05
CD3^+^T cells, μL	736.00(476.00, 1044.00)	684.00(432.00, 1038.00)	758.00(506.00, 1058.00)	>0.05
CD4/CD8 ratio	1.85(1.39, 2.39)	1.81(1.38, 2.36)	1.95(1.42, 2.44)	>0.05
WBC, 10^9^/L	13.21 ± 8.99	13.27 ± 4.67	13.16 ± 11.60	>0.05
NEU, 10^9^/L	11.21 ± 6.48	11.38 ± 4.18	11.06 ± 8.02	>0.05
LYM, 10^9^/L	1.12 ± 0.82	1.15 ± 0.75	1.09 ± 0.88	>0.05
PLT, 10^9^/L	194.57 ± 87.01	196.10 ± 88.08	193.18 ± 86.36	>0.05
CRP, mg/L	76.31 ± 72.00	79.55 ± 76.06	73.39 ± 68.32	>0.05
PCT, ng/ml	0.56(0.13, 1.87)	0.76(0.19, 1.99)	0.42(0.12, 1.83)	>0.05
ALB, g/L	31.81 ± 6.76	30.89 ± 5.80	32.63 ± 7.44	0.043
Lactate, mmol/L	1.70(1.10, 3.20)	2.00(1.20, 3.20)	1.30(0.98, 3.38)	0.011

GCS,Glasgow Coma Scale; APACHE II score, Acute Physiology and Chronic Health Evaluation II score; IL-10, Interleukin-10; IL-17A, Interleukin-17A;IL-6, Interleukin-6; WBC, White blood cell; NEU, Neutrophil; LYM, Lymphocyte; PLT, Platelet; CRP, C-reactive protein; PCT, Procalcitonin; ALB, Albumin.

### 3.2 Characteristics of infectious pathogens

Among the 117 patients in the MDRO group, 142 multidrug-resistant strains were isolated, predominantly Gram-negative bacteria. These included: carbapenem-resistant Acinetobacter baumannii (CRAB) (79/142, 55.63%); extended-spectrum beta-lactamase-producing Klebsiella pneumoniae (ESBL-KP) (31/142, 21.83%); carbapenem-resistant K. pneumoniae (CRKP) (1/142, 0.70%); and carbapenem-resistant Pseudomonas aeruginosa (CRPA) (21/142, 14.79%). The only Gram-positive pathogen was methicillin-resistant Staphylococcus aureus (MRSA) (10/142, 7.04%). Additionally, 72 non-resistant strains were identified in the MDRO group, with common isolates being K. pneumoniae (n=25, 34.72%), P. aeruginosa (n=15, 20.83%), S. aureus (n=9, 12.50%), and Stenotrophomonas maltophilia (n=8, 11.11%). Fungal co-infection was present in 44 patients (37.60%) in this group. In the non-MDRO group, no pathogen was detected in 73 patients (56.15%). Among the remaining patients, a total of 71 bacterial strains were isolated, with common isolates being K. pneumoniae (n=25, 35.21%), P. aeruginosa (n=20, 28.17%), and S. aureus (n=6, 8.45%). Fungal co-infection was identified in approximately 39 patients (30.00%).

### 3.3 Correlations between the IL-10, IL-6 and MDRO risk factors

As shown in **Table 2**, we analyzed three multivariable logistic regression models to assess the associations between IL−10, IL−6, and MDRO infection.IL−10 showed a significant positive association with MDRO risk, and this association remained robust after incremental adjustment for confounders. Across all three models, IL−10 was significantly and positively associated with the risk of MDRO infection (Model 1: OR = 5.00, 95% CI: 2.56–9.75; Model 2: aOR = 5.18, 95% CI: 2.62–10.26; Model 3: aOR = 4.45, 95% CI: 2.16–9.17; *P* < 0.001). Compared with the lowest tertile (T1), the highest tertile (T3) of IL−10 was associated with a significantly increased risk of MDRO infection in all models (*P* for trend < 0.001). These results indicate that higher IL−10 levels correspond to a greater risk of MDRO infection, demonstrating a clear dose−response relationship that persisted after adjustment for common confounders. In contrast, the association between IL−6 and MDRO risk was weaker. While not significant in the unadjusted model, each SD increase in IL−6 was associated with a 66% higher odds of MDRO infection in the partially adjusted model (aOR = 1.66, 95% CI: 1.02–2.68, *P* = 0.040) and a 52% increase in the fully adjusted model (aOR = 1.52, 95% CI: 1.01–2.28, *P* = 0.042). However, analysis by IL−6 tertiles showed no statistically significant association in the incrementally adjusted models.

**Table 2:** Associations between IL-10, IL-6 and the risk of MDRO infection.

Variables	Model 1		Model 2		Model 3	
	OR (95%CI)	*P* value	OR (95%CI)	*P* value	OR (95%CI)	*P* value
IL-10 Per SD increase	5.00(2.56-9.75)	<0.001	5.18(2.62-10.26)	<0.001	4.45(2.16-9.17)	<0.001
IL-6 Per SD increase	1.59(0.99-2.55)	>0.05	1.66(1.02-2.68)	0.040	1.52(1.01-2.28)	0.042
IL-10						
T1	Ref.		Ref.		Ref.	
T2	2.69 (1.40-5.31)	0.004	3.07 (1.55-6.24)	0.002	2.72 (1.26-6.08)	0.012
T3	8.73 (4.41-17.98)	< 0.001	9.67 (4.78-20.53)	< 0.001	9.69 (4.27-23.35)	< 0.001
*P* for trend	<0.001		<0.001		<0.001	
IL-6						
T1	Ref.		Ref.		Ref.	
T2	1.07 (0.58-2)	0.823	1.17 (0.62-2.23)	0.619	0.84 (0.40-1.75)	0.645
T3	2.03 (1.1-3.81)	0.025	2.36 (1.24-4.59)	0.010	1.40 (0.63-3.09)	0.408
*P* for trend	0.025		0.010		0.417	

Model 1 was unadjusted; Model 2 was adjusted for sex, age, diabetes, smoking status and alcohol consumption; Model 3 was further adjusted for surgical status, primary neurological disease, length of ICU stay, prior antibiotic use, GCS, APACHE II score, duration of mechanical ventilation, CRP, ALB, PCT and lactate.

MDRO, multidrug-resistant organism; OR, Odds Ratio; CI, confidential interval; GCS,Glasgow Coma Scale; APACHE II score, Acute Physiology and Chronic Health Evaluation II score;IL-10, Interleukin-10;IL-6, Interleukin-6; CRP, C-reactive protein; PCT, Procalcitonin; ALB, Albumin.

### 3.4 Subgroup analysis

**Figure 2:**
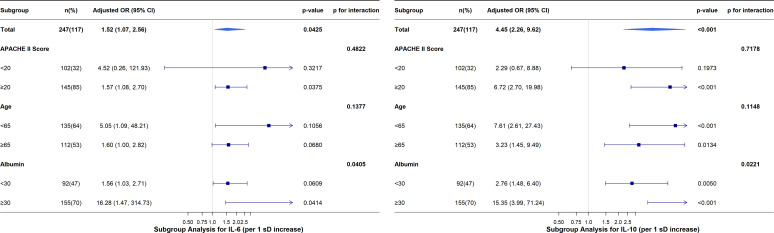
Subgroup and interaction analysis for IL−10 and IL−6 (per SD) in relation to MDRO across various subgroups.

Exploratory subgroup analyses were performed based on age, albumin level, and APACHE II score (**Figure 2**). A significant interaction was observed for albumin level (*P* for interaction < 0.05 for both cytokines). Notably, the positive associations between IL-10, IL-6 and MDRO risk were substantially stronger in patients with normal albumin levels (≥ 30 g/L). No significant associations were observed in the other two subgroups (age and APACHE II score).

### 3.5 Evaluation of the predictive value of IL−10 and IL−6 for MDRO infection risk

**Figure 3:**
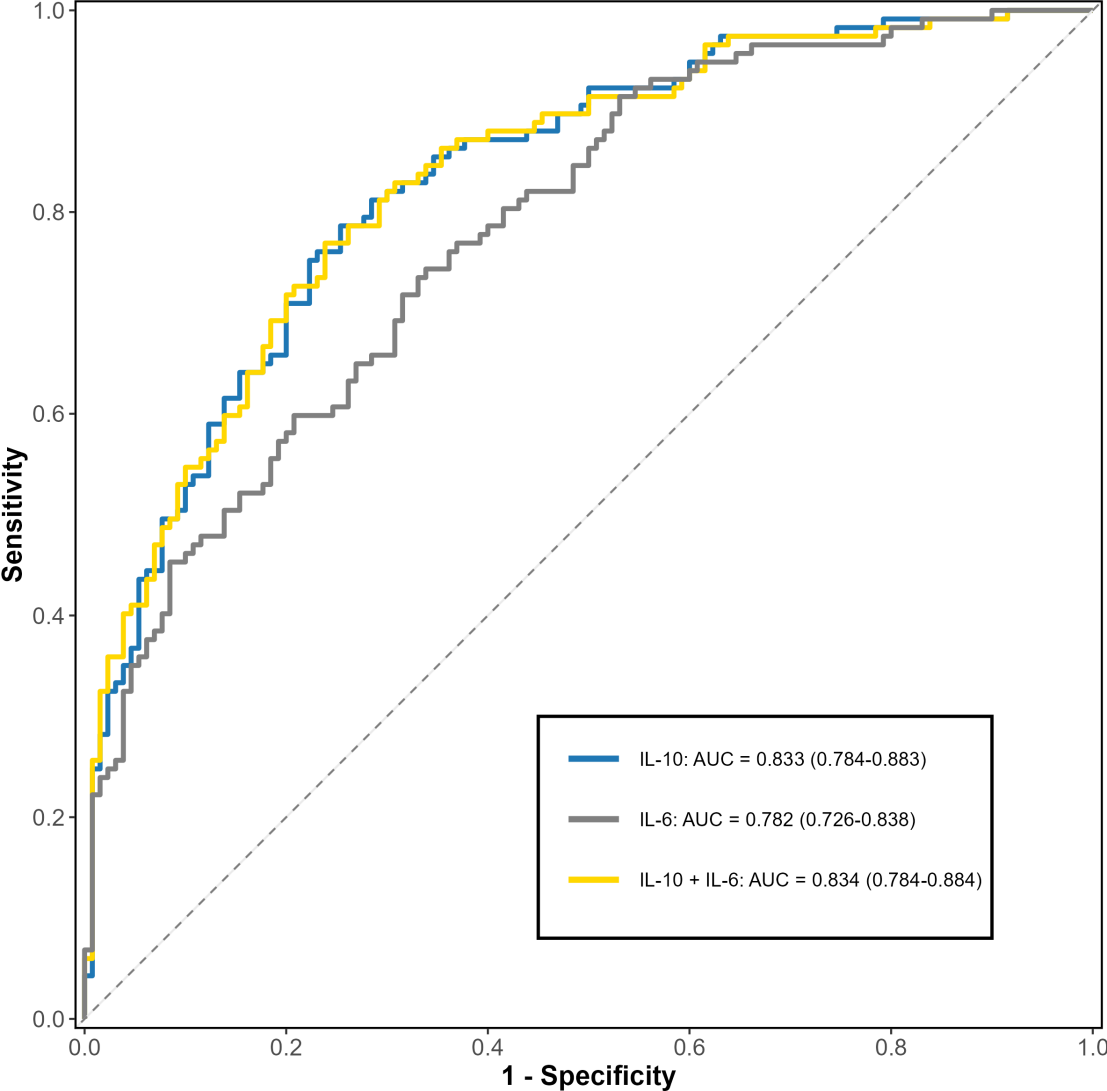
ROC curves of IL-10 and IL-6 in predicting MDRO infection risk.

As shown in **Figure 3**, IL-10, IL-6, and their combined model (IL-10 + IL-6) all demonstrated good diagnostic performance for MDRO infection, with areas under the curve (AUCs) of 0.833 (95% CI: 0.784 – 0.883), 0.782 (95% CI: 0.726 – 0.838), and 0.834 (95% CI: 0.784 – 0.884), respectively. In **Table 3**, the diagnostic efficacy of IL-10 was significantly higher than that of IL-6 (*P* for comparison = 0.003). However, the combined model (IL-10 + IL-6) did not show a statistically significant improvement over IL-10 alone (*P* for comparison = 0.842). At its optimal cutoff value of 0.427, the IL-10 model achieved a sensitivity of 78.6% and a specificity of 74.6%.

**Table 3:** The predictive value of IL-10 and IL-6 for MDRO infection risk.

Variables	AUC (95% CI)	*P* value	*P* for comparison	Optimal cutoff value	Sensitivity	Specificity	Youden index
IL-10	0.833 (0.784-0.883)	<0.001	Ref.	0.427	0.786	0.746	0.532
IL-6	0.782 (0.726-0.838)	<0.001	0.003	0.416	0.744	0.662	0.405
IL6+IL10	0.834 (0.784-0.884)	<0.001	0.842	0.439	0.769	0.762	0.531

MDRO, multidrug-resistant organism; AUC, area under the curve; CI, confidence interval; IL-10,Interleukin-10; IL-6,Interleukin-6.

### 3.6 Comparison of IL-10 and IL-6 levels among patients in the six pathogen subgroups.

Serum levels of IL-10 and IL-6 across the six patient groups are presented in **Table 4**. The Kruskal−Wallis test revealed statistically significant overall differences among the six groups for both IL-10 (*P* < 0.001) and IL-6 (*P* = 0.007).

**Table 4:** Comparison of IL-10 and IL-6 levels in patients grouped by six pathogen subgroups.

Variables		n (%)	IL-10 (pg/mL)	IL-6 (pg/mL)
MDRO	CRAB	58(24.1)	5.47(3.92, 7.76)	26.85(14.86, 95.39)
	ESBL-KP	21(8.8)	5.87(4.88, 7.24)	87.75(29.69, 334.55)
	CRPA	10(4.2)	7.23(4.77, 10.85)	83.79(58.11, 158.06)
	MDRO mixed infection	21(8.8)	4.86(3.91, 8.78)	39.19(12.01, 94.47)
non-MDRO	culture-positive	57(23.8)	3.24(2.16, 5.25)	28.21(12.92, 74.66)
	culture-negative	73(30.4)	3.04(1.80, 3.91)	31.27(13.05, 61.87)
Overall *P*-value			<0.001	0.007

CRAB, carbapenem-resistant Acinetobacter baumannii; ESBL-KP, extended-spectrum beta-lactamase-producing Klebsiella pneumoniae; CRPA, Carbapenem-resistant Pseudomonas aeruginosa; MDRO, multidrug-resistant organism; IL-10,Interleukin-10; IL-6,Interleukin-6.

**Figure 4:**
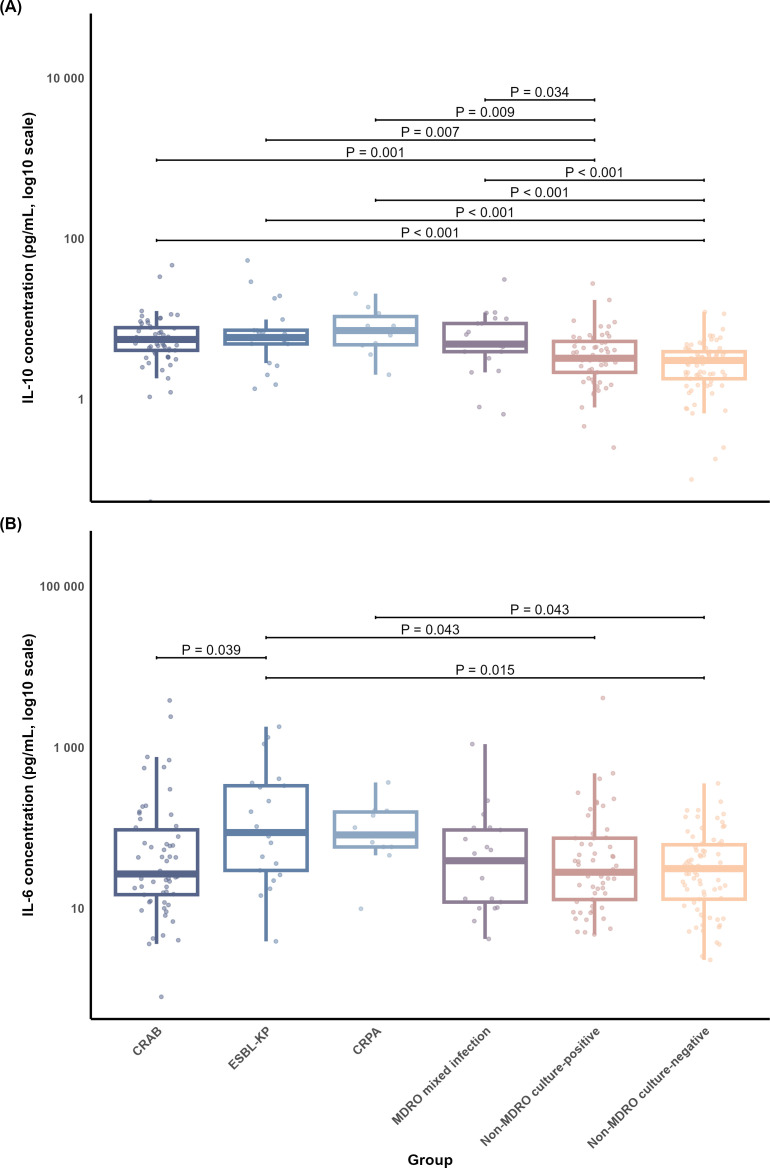
Boxplots comparing IL-10 **(A)** and IL-6 **(B)** concentrations among the six pathogen subgroups, with results of Dunn’s *post-hoc* test. Two boxplots are presented to illustrate the median (horizontal line within each box) and interquartile range (IQR, box) of IL-10 **(A)** and IL-6 **(B)** concentrations across the six pathogen-stratified groups. Post-hoc pairwise comparisons were performed using Dunn’s test, with original P-values adjusted by the Benjamini-Hochberg method (false discovery rate, FDR). In the figure, black lines connecting groups indicate pairs with an adjusted P < 0.05, and the corresponding adjusted P-values are displayed above the lines. CRAB, carbapenem-resistant Acinetobacter baumannii; ESBLKP, ESBL-producing Klebsiella pneumoniae; CRPA, Carbapenem-resistant Pseudomonas aeruginosa; MDRO, multidrug-resistant organism; IL-10, Interleukin-10; IL-6, Interleukin-6.

Boxplots illustrating the levels of these two immune factors, along with the results of Dunn’s post−hoc test, are shown in **Figure 4**. Among pairwise comparisons within the four MDRO−related groups (CRAB, ESBL−KP, CRPA, and mixed MDRO infection), a statistically significant difference was observed only for serum IL-6 levels between the CRAB and ESBL−KP groups (adjusted *P* = 0.039, Benjamini-Hochberg), whereas serum IL-10 levels did not differ significantly across these four subgroups. Notably, all four MDRO subgroups exhibited significantly higher IL-10 levels compared to both the non−MDRO culture−positive and non−MDRO culture−negative groups (all adjusted *P* < 0.05,Benjamini-Hochberg). Regarding IL-6, significantly higher levels were observed only in the ESBL−KP and CRPA subgroups when compared to the non−MDRO culture−positive and culture−negative groups.

## 4 Discussion

Most existing studies have focused on populations with well-defined immunosuppressive conditions, such as tumors, HIV, hematological malignancies, or autoimmune diseases (32–34). In our study, we excluded patients with these known immunosuppressive disorders or other conditions that could significantly perturb immune factors. In both groups, the mean or median values of T-cell parameters, including CD4^+^ T cells, CD8^+^ T cells, CD3^+^ T cells, and the CD4/CD8 ratio, fell within the laboratory reference intervals, and univariate analysis showed no statistically significant differences, indicating that the two groups had no severe intrinsic immune defects. Furthermore, baseline inflammatory burden indicators collected on day 2 of mechanical ventilation also showed no significant differences between the groups, suggesting that inflammation induced by mechanical ventilation itself was comparable. Theoretically, this represents a more homogeneous cohort. However, this does not imply that T lymphocytes play no role in infection. Substantial differences exist between pneumonia in immunocompromised hosts and pneumonia in non-immunocompromised hosts, encompassing baseline immune reserve, post-infection immune activation, levels of immune cytokines, feedback regulation of inflammatory responses, and the processes of inflammation resolution and tissue repair. HIV infection depletes CD4^+^ T-helper cells, reducing the host’s baseline immune cell reservoir and dampening cytokine production, thereby attenuating the inflammatory response (35, 36). The immune network mechanisms in pneumonia patients with cancer or autoimmune diseases are more complex (37), which can affect the development and recovery from pneumonia. In MDRO-infected patients with relatively intact immune function, a robust inflammatory response can be initiated to clear pathogens. The core of their disrupted immune homeostasis lies in an imbalance between pro-inflammatory and anti-inflammatory cytokines, rather than an absolute deficiency of immune cells.

Our study found that serum IL-6 and IL-10 levels were significantly elevated in the MDRO group and remained independent risk factors after full adjustment (**Table 2**). The combined IL-10+IL-6 model showed good predictive performance (**Figure 3**), suggesting that monitoring these cytokines may aid early identification of MDRO infection in VAP patients. The above findings may reflect an immune dysregulation state that occurs as the body attempts to repair itself in response to tissue injury, external pathogen invasion, and iatrogenic mechanical stimulation. First, this concurrent elevation of pro-inflammatory and anti-inflammatory cytokines may result from the combined effects of Th17/Tregs imbalance and immune feedback regulation (38). Th17 cells primarily produce IL-17A, which stimulates the expression of pro-inflammatory cytokines such as IL-6 and TNF-α, while Tregs produce IL-10. During the initial phase of pneumonic inflammation, the immune system amplifies the inflammatory response via positive feedback (39). Innate immune cells secrete factors such as IL-6 and TGF-β, which promote Th17 differentiation while inhibiting Treg generation (40), in order to promote inflammation and resist pathogen invasion. A study has demonstrated that dysbiosis of the respiratory microbiota (e.g., Klebsiella pneumoniae) can promote the pathogenicity of Th17 cells and the progression of central nervous system inflammation (41). Th17 cells are responsible for inflammatory responses; excessive activation of Th17 cells can induce a cytokine storm, leading to an elevated Th17/Tregs imbalance, which is associated with the severity of pneumonia and accelerates disease progression (42). Furthermore, a key positive feedback amplification loop exists between IL-6 and IL-17A: IL-6 promotes the differentiation of naive T cells into Th17 cells and enhances IL-17A production (43), while IL-17A acts on various stromal cells such as fibroblasts and epithelial cells to further increase IL-6 expression and secretion, thereby elevating serum IL-6 levels (44). The above evidence indicates that IL-17A and IL-6 have synergistic pro-inflammatory effects, and the elevated IL-6 observed in this study reflects the activation of Th17 responses and sustained activation of the positive feedback loop at the serum level. It is worth noting that the majority of IL-17A concentrations in this study fell below the detection limit of 10 pg/mL. This may indicate that circulating IL-17A levels in VAP patients are extremely low (45), and the stability of IL-17A detection is significantly affected by sample handling and storage conditions (46), making it unsuitable as a biomarker. In contrast, the stability of IL-6 under routine clinical testing conditions is more widely recognized (47), it reflects the overall inflammatory burden, and it is routinely detectable, making it more suitable for routine assessment. Therefore, the early phase of MDRO infection (within 72 hours) is a critical period for inflammation amplification and immune injury formation. Elevated IL-6 levels reflect the high pathogenicity and inflammatory burden of MDRO. When IL-6 is markedly elevated, clinicians should be vigilant for disease progression and possible MDRO infection, and it may help in assessing early empirical anti-MDRO therapy and corticosteroid anti-inflammatory treatment.

As inflammation progresses, negative feedback mechanisms engage, and activated regulatory T cells and macrophages produce abundant IL-10, which suppresses excessive inflammation and prevents tissue damage (48, 49). Qin Y et al. showed that in the early stage of infection, Tregs exert anti-inflammatory functions by secreting anti-inflammatory cytokines and expressing key co-inhibitory molecules; however, overexpression of Tregs in the late stage of infection may increase susceptibility to secondary infections (50). This finding is consistent with the elevated IL-10 levels observed in our study, reflecting that the increase in IL-10 during the early stage of MDRO infection represents a compensatory activation of negative feedback regulation. Overexpression of Tregs may lead to Th17/Tregs imbalance. In addition, maintaining Th17/Treg balance may provide protective immunity against pulmonary infection (51). The combined action of IL-17A and IL-10 enables the host to control infection without causing excessive inflammatory tissue damage (52). Studies have also shown that modulating the balance of cytokines in lung tissue and serum (reducing IL-6 and IL-17A while increasing IL-10) is a consequence of protective immunomodulation following infection (53). The above evidence is consistent with the findings of our study, which show that a simultaneous elevation of IL-6 and IL-10 is an immune feature in the early stage of MDRO infection during VAP. Due to the positive feedback loop involving IL-6, Th17, and IL-17A, the elevated levels of IL-6 and IL-10 indicate, to some extent, that both Th17 and Treg cells are overactivated or dysfunctional, leading to Th17/Treg imbalance. This reveals that early MDRO infection in VAP induces a more pronounced pro-inflammatory and anti-inflammatory synchronous activation pattern compared to that triggered by susceptible bacteria, which may serve as a quantifiable and simple immunotyping tool. Therefore, when a simultaneous marked elevation of IL-10 and IL-6 is detected in the early stage of VAP, clinicians should be alerted to a high risk of MDRO infection and take timely antibiotic intervention. This approach may facilitate a shift in the diagnosis and treatment of infectious diseases from a “pathogen-oriented” strategy toward a “host-pathogen co-targeted” model.

Second, the underlying neurocritical illness itself, sustained mechanical ventilation stimulation, and gut-microbiome-brain axis dysregulation also contribute to the process of host immune dysregulation. In the early stage of severe neurological disorders (e.g., traumatic brain injury, intracranial hemorrhage), a robust immune-inflammatory response is triggered, characterized by a dynamic balance between IL-6-driven pro-inflammation and IL-10-mediated anti-inflammatory compensation (9). Multiple studies have shown that the mechanical stimulation of ventilation can induce the production and secretion of inflammatory cytokines such as IL-6, IL-8, and IL-10 (54, 55). Mairi Ziaka et al. suggested that a state of multi-source immune dysregulation may result from the combination of acute brain injury (ABI), mechanical ventilation-induced mechanical stimulation, and gut-microbiome-brain axis dysregulation (56). Therefore, we propose that the concurrent elevation of IL-10 and IL-6 in VAP patients may indicate a state of multi-source immune dysregulation and Th17/Treg imbalance, and may suggest a high risk of MDRO infection. A comprehensive assessment of patient condition by combining the inflammatory-immune status (elevated IL-10 and IL-6) with other clinical indicators (such as APACHE II score, GCS score, and albumin) can facilitate timely initiation of appropriate anti-infective therapy covering MDRO, so as to interrupt the vicious cycle of “inflammation-immune dysregulation-persistent infection.” Moreover, adjunctive supportive measures including regulating Th17/Treg imbalance, optimizing lung-protective ventilation strategies, and protecting gut microbiota may help improve the prognosis of patients with MDRO infection.

Additionally, the results of our subgroup analyses stratified by age, albumin level, and APACHE II score (**Figure 2**) showed that among patients with relatively normal albumin levels (≥30 g/L), both high IL-10 and high IL-6 were associated with substantially elevated MDRO risk (aORs 15.35 and 16.28, respectively), with effect sizes markedly greater than those in patients with hypoalbuminemia. This finding appears contrary to conventional medical knowledge and may suggest that in hosts with relatively normal albumin levels, marked elevations of cytokines (whether pro−inflammatory or anti-inflammatory) could signal a concealed and profound dysregulation of the immune internal environment. Therefore, in clinical practice, it is crucial to interpret cytokine levels in the context of a patient’s albumin status. For patients with normal albumin and elevated cytokine levels, even if they appear “well-nourished,” heightened vigilance for MDRO risk is warranted, necessitating more proactive microbiological surveillance and infection-prevention interventions.

Based on the available data and methods in this study, the results of subgroup analyses showed (**Figure 4**) that serum IL-10 levels did not differ significantly among the four MDRO subgroups (CRAB, ESBL-KP, CRPA, and mixed MDRO infection). However, all four MDRO subgroups exhibited significantly higher IL-10 levels compared to both the non-MDRO culture-positive and culture-negative groups. This finding largely suggests that early IL-10 release in VAP patients may represent a universal host immune response to multidrug-resistant organism infection, which is not substantially influenced by specific pathogen types, and its serum level may sensitively reflect the host’s immune dysregulation status.

In contrast, among the MDRO subgroups, serum IL-6 levels were significantly elevated in patients infected with ESBL-KP and CRPA (**Table 4**). Notably, as shown in **Figure 4**, serum IL-6 levels were significantly higher in patients with ESBL-KP infection compared to those with CRAB infection (adjusted *P* = 0.039, Benjamini-Hochberg), demonstrating pathogen specificity. This heterogeneity suggests that a baseline state of elevated IL-6 may be driven by specific MDRO infections. From an alternative perspective, considering IL-6 as a pro-inflammatory cytokine, its increased levels in non-immunocompromised hosts likely reflect an active immune response to infection, which may be particularly induced by ESBL-KP strains. It has been reported that non-hypervirulent Klebsiella pneumoniae induces high expression of Th17 cytokines (IL-23, IL-1β, and IL-6) (57). Elevated IL-6 can increase the survival rate of mice with pulmonary Klebsiella infection and enhance bacterial clearance (58). Further research is warranted to elucidate the immune response specifically associated with ESBL-KP strains. An alternative explanation is that CRAB may possess a weaker capacity to induce IL-6 production, or may even exhibit a defect in its pro-inflammatory potential, resulting in a relatively suppressed state. One possible mechanism is that CRAB may modulate the host inflammatory response through multiple pathways. Yang Yang et al. reported that CRAB may activate the cGAS-STING pathway via the outer membrane protein OMP38, inducing a type I interferon response that paradoxically favors bacterial survival, thereby leading to reduced expression of classical pro-inflammatory cytokines such as IL-6 (59). Another study showed that repeated pulmonary A. baumannii infection in mice induced tolerance, with high expression of Tregs establishing an immunosuppressive environment and leading to low levels of IL-6 and TNF-α in both bronchoalveolar lavage fluid (BALF) and plasma (60). Additionally, this observed reduction in IL-6 levels may be stage-specific or site-specific, for instance, manifesting within the pulmonary microenvironment.

However, there are spatial differences in cytokine levels between serum and lung tissue. As a systemic inflammatory marker, IL-6 is stable and readily detectable in serum. IL-10 represents the combined production from local tissues, the spleen, and circulating immune cells, and its serum level increases with a lag behind local levels (61, 62). IL-17A primarily acts locally, with low and highly variable serum levels (63). This study measured only serum cytokines and did not collect lung tissue samples; therefore, the levels do not directly reflect the pulmonary inflammatory milieu. Although serum IL-17A levels did not differ significantly between the MDRO and non-MDRO groups, its local effects should not be overlooked. This study lacks dynamic monitoring data, which may result in the loss of critical information; only serial monitoring (e.g., repeated measurements over time) can more accurately capture the true evolution of cytokine profiles during the infectious process. Patients with secondary bloodstream infection (BSI) were not excluded from this study, which may theoretically have some impact on cytokine levels; however, a sensitivity analysis recalculated after excluding patients with BSI showed that the association between IL‑10 and MDRO infection remained robust, and the IL‑6/IL‑10 ratio was unaffected (**Supplementary Table 1**). Furthermore, all findings in this study are based on a single-center retrospective study. There may be unmeasured confounding factors that could affect the reliability of the results, and these findings have not been validated in an independent cohort. Whether these results can be generalized to broader populations requires further investigation.

## 5 Conclusion

In summary, elevated serum levels of IL-10 and IL-6 within 72 hours after the onset of VAP in non-immunocompromised patients are early immunological markers of MDRO infection, with IL-10 showing potential as an independent biomarker. Their concurrent elevation reflects a state of pro-inflammatory and anti-inflammatory immune imbalance caused by multiple factors. Combined assessment with other clinical indicators (e.g., albumin) may guide individualized early anti-infection therapy. IL-10 lacks specific strain discrimination ability but sensitively reflects immune dysregulation, whereas IL-6 exhibits pathogen specificity and may aid in identifying ESBL-KP/CRPA infection. These findings provide theoretical insights into cellular immune imbalance, identify potential targets for immunomodulation, and facilitate individualized early identification and treatment of MDRO infection.

## Data Availability

The raw data supporting the conclusions of this article will be made available by the authors, without undue reservation.
