# Supplementary material for: Immune imbalance markers: key factors in early recognition of multidrug-resistant bacterial infections in non-immunocompromised VAP patients
Source: Front Immunol. 2026 Jun 4;17:1799042. doi: 10.3389/fimmu.2026.1799042 (PMC13275356; doi:10.3389/fimmu.2026.1799042)
Supplement: Supplementary file 1 [file Table1.docx]

Comparison of serum IL-6 and IL-10 levels (median, IQR) and their association with MDRO infection (crude odds ratio per 1-SD increase, 95% CI from univariate logistic regression) in the original cohort (N=247) and after excluding patients with secondary bloodstream infection (BSI) (N=233).

| **Variables** | **MDRO status** | **N** | **IL-6 (pg/mL)** | **IL-10 (pg/mL)** | **OR per 1-SD increase (95% CI)** |
| --- | --- | --- | --- | --- | --- |
| **Original** | No | 130 | 29.39  （12.72，63.57） | 3.11  （1.82，4.42） | IL-6: 1.59(0.99-2.55)  IL-10:5.00(2.56-9.75) |
|  | Yes | 117 | 43.99  （15.93，146.90） | 5.55  (3.89，8.78) |  |
| **Excluding BSI** | No | 126 | 29.39  (12.36，63.41) | 3.16  (1.99，4.45) | IL-6: 1.40 (1.01-2.42)  IL-10:4.47 (2.42-9.04) |
|  | Yes | 107 | 43.99  (15.93，136.16) | 5.62  (3.80，8.48) |  |

Comparison of serum IL-6/IL-10 ratios between the original cohort (N=246) and the cohort after excluding patients with secondary bloodstream infection (BSI) (N=232).

| **Variables** | **Original**  **(N=246)** | **Excluding BSI**  **(N=232)** | **P-value** |
| --- | --- | --- | --- |
| **IL-6/IL-10 ratio** | 10.17（4.23，22.33） | 9.70（4.10，21.93） | 0.80 |

Note: One patient from the original cohort of 247 had an IL-10 value of “0” and was excluded from ratio calculation, leaving N=246. The BSI-excluded cohort further excluded patients with secondary bloodstream infection (N=14), resulting in N=232.
